# Pediatric Cancers: Insights and Novel Therapeutic Approaches

**DOI:** 10.3390/cancers15143537

**Published:** 2023-07-08

**Authors:** Saurabh Agarwal

**Affiliations:** Department of Pharmaceutical Sciences, College of Pharmacy and Health Sciences, St. John’s University, New York, NY 11439, USA; agarwals@stjohns.edu ; Tel.: +1-718-990-1623

Pediatric cancers cast a dark shadow over the lives of countless children and their families and represent a leading cause of mortality among children worldwide. Despite significant advancements in medical research and treatment modalities, the battle against pediatric cancers remains a challenging and urgent priority. Every day, numerous children are diagnosed with cancers such as leukemia, brain tumors, neuroblastoma, sarcomas, and others, thrusting them into a grueling battle against an invisible enemy. Treatments of pediatric cancers present unique challenges compared to adult cancers, due to the harsh side effects of chemotherapy and radiation, and delayed diagnosis. It is important to understand the unique challenges and insights of different pediatric cancers and to develop novel therapeutic approaches. This editorial covers the unique special edition on Pediatric Cancers published in *Cancers*, which consists of 24 articles presented by international leaders in the pediatric cancers field. This Special Issue comprises 16 original research articles, 6 reviews, and 2 systematic reviews covering insights on different pediatric cancers.

Conventional chemotherapies play a vital role in pediatric cancer treatment, especially in low- and middle-income countries. Several chemo drugs have been approved by the FDA for pediatric cancers; however, multi-drug resistance (MDR) and transporters mediating MDR pose serious obstacles to drug efficacy and require dose escalation, which leads to side effects in pediatric patients [1]. The review by Bo et al. summarizes the mechanisms involving MDR and different drug transporters found in pediatric tumors. Understanding the mechanisms of MDR transporters will enhance the efficacy of pediatric chemotherapies to improve overall survival and reduce treatment toxicity [1]. A systematic review of the effect of chemotherapy in the management of a very rare pediatric neoplasm Melanotic Neuroectodermal Tumor of Infancy (MNTI) highlights the effects of chemotherapy treatments in supporting surgical resections in inoperable, metastatic, and recurrent cases of MNTI [2]. Another retrospective study showed substantial infectious morbidity in pediatric sarcomas patients during neoadjuvant chemotherapy treatment [3]. This study emphasizes developing better risk stratification protocols for preventing and managing febrile neutropenia and infections to maintain quality of life and better chemotherapy treatment outcomes [3]. Understanding the interrelationships between childhood cancers and their treatment with chronic stress in patients throughout their lifespan is very important for effectively managing survivorship. White et al. review the physical, neurological, and psychological effects that lead to chronic stress in childhood cancer survivors and advocate for effective stress management for overall better outcomes [4].

The most common childhood cancer is acute leukemia, which accounts for almost 28% of all pediatric cancers. The most common types in children are acute lymphocytic leukemia (ALL) and acute myeloid leukemia (AML) [5]. An acute leukemia data study of 690 patients showed that the incidence of hyperleukocytosis was 16.6% in ALL and 20.3% in AML patients and was associated with higher morbidity rates and worse survival outcomes. This study suggests modifying the treatment regimen and improving the early-stage monitoring [5]. Myeloid sarcomas (MS), commonly referred to as chloromas, are extramedullary tumors of AML with varying incidence and influence on outcomes [6]. The review article by Zorn et al. summarizes the current understanding of pediatric MS and its biological drivers to spark the development of effective therapeutic strategies for MS and AML [6].

The second most common pediatric cancers are brain and spinal cord tumors, accounting for about 26% of childhood cancers. There are many types of brain and spinal cord tumors, and the treatment and pathogenesis for each are different. The review article by Thorbinson and Kilday summarizes the molecular landscape, prognosis, current therapies, and novel therapeutic approaches for the three most common pediatric brain tumors medulloblastoma, high-grade gliomas, and ependymoma [7]. Further, Chen et al. review the immune microenvironment and immunotherapy clinical trials for the diffuse intrinsic pontine glioma (DIPG) [8]. This glial tumor, DIPG, accounts for 10–15% of pediatric brain tumors, with no effective treatment in the clinic. Therefore, developing novel immunotherapies for transforming DIPG tumors from cold to hot holds a high potential for effective DIPG treatment [8]. Ependymoma is the third most prevalent pediatric CNS tumor with considerable molecular and clinical diversity. The review by Zaytseva et al. underscores the importance of comprehensive molecular profiling to identify (epi)genetic variants for advanced risk stratification of patients [9]. This profiling will support better management and will be pivotal for the development of novel therapeutic strategies for ependymoma [9].

The third most common pediatric cancer is neuroblastoma (NB) which accounts for almost 15% of all pediatric cancer deaths with an overall 5-year survival rate of less than 50% [10]. Therefore, developing novel therapeutic approaches targeting the molecular mechanisms that drive NB progression is very important. In this Special Issue, we have published six research articles on developing effective therapeutic strategies for NB using in vitro and in vivo tumor models. A dual HDAC and PI3K inhibitor CUDC-907 [10] and an ERK inhibitor Ulixertinib [11] were found to be effective treatment approaches for high-risk NB. Further, SOX4 is shown to mediate NB cell differentiation, and SOX4 knockdown partially reversed retinoic acid-induced differentiation in NB cells [12]. The LPA-LPAR1 axis is shown to have migration-inhibitory effects on NB cells, and knockdown of LPRA1 promotes NB cell migration and metastasis [13]. The featured paper published in this Special Issue researches the role of Serine-threonine kinase receptor-associated protein (STRAP) in NB [14]. Bownes et al. found that the genetic knockout of STRAP inhibits NB cell proliferation in vitro and tumor growth in vivo, and overall concluded that STRAP plays a role in NB stemness and tumorigenesis [14]. A retrospective clinical study in 217 high-risk NB patients found that oral metronomic maintenance treatment can improve overall survival in patients not treated with autologous stem cell transplantation or anti-GD2 antibody therapy [15].

Nephroblastoma or Wilms tumor is the most common kidney tumor in childhood and accounts for almost 5% of all pediatric cancers [16]. A retrospective clinical study in 2927 Wilms tumor patients advocates for considering cancer predisposition syndrome with causative genetic or epigenetic variants for effective genetic counseling and management [16]. Pediatric sarcomas represent a diverse group of rare bone and soft tissue malignancies comprising almost 13% of all pediatric cancers [17]. The most common bone sarcomas are osteosarcomas and Ewing’s sarcoma, while rhabdomyosarcoma is the most common soft-tissue pediatric sarcoma [17]. A clinical study of 620 survivors of pediatric bone sarcomas in Nordic countries reveals that these patients are at increased risk of developing somatic diseases and long-term adverse health effects [18]. This study emphasizes intervention strategies for optimal patient counseling and follow-up care for the survivors of osteosarcoma and Ewing’s sarcoma [18]. Kim et al. developed a radio-genomics predictive model that incorporates both imaging features and gene expression to accurately predict metastasis and chemotherapy responses to improve pediatric osteosarcoma patient outcomes [19]. Chen et al. identified RNA-binding protein level patterns as a prognostic model to identify Ewing’s sarcoma patients with a higher mortality risk [20]. An interesting research study screening natural product extracts identified fungal metabolite altertoxin II (ATXII) inhibiting Ewing’s sarcoma growth both in vitro and in vivo [21]. ATXII was found to induce DNA double-strand breaks and cell cycle S phase accumulation without directly binding to the DNA [21]. In rhabdomyosarcoma, increased EZH2 protein levels are associated with poor prognosis and increased metastatic potential [22]. Direct and indirect targeting of EZH2 showed differential efficacy due to divergent epigenetic and cellular signaling regulations in different rhabdomyosarcoma cell subtypes [22]. A research study by Lavoie et al. using cell surface proteomics, transcriptomics, and tissue specimens uncovers B7-H3 as a major immunoregulatory molecule expressed by rhabdomyosarcomas and not by normal human tissues [23]. Furthermore, B7-H3 knockout in rhabdomyosarcoma tumor cells increases T-cell-mediated cytotoxicity, indicating B7-H3 as a potential target for developing next-generation immunotherapies for rhabdomyosarcoma [23]. Fuchs et al. used meta-analysis data and suggest developing a uniform classification for pediatric rhabdoid liver tumors and small cell undifferentiated liver tumors, due to the evidence of overlapping histopathology and significantly better survival [24]. Desmoplastic small round cell tumor (DSRCT) is a rare and aggressive soft tissue sarcoma, characterized by a chromosomal translocation resulting in the *EWSR1-WT1* gene fusion [25]. Blejis et al. develop a novel primary patient-derived DSRCT in vitro model recapitulating the original tumor to study disease progression and drug sensitivity [25].

Overall, this Special Issue on pediatric cancers brings together the current understanding of different pediatric cancers, their management, sub-types, and molecular features. Additionally, this Special Issue also includes research articles on experimental therapeutics for different pediatric cancers. In conclusion, this Special Issue book will provide the readership with a comprehensive knowledge of pediatric cancers and hope for developing more effective and less-toxic therapeutic strategies for children battling with cancers.
